# Electroencephalographic evaluation under standing sedation using sublingual detomidine hydrochloride in Egyptian Arabian foals for investigation of epilepsy

**DOI:** 10.1111/jvim.16695

**Published:** 2023-04-07

**Authors:** Tatiana Vinardell, Sami Elestwani, Camilla Jamieson, Ejaz Karim, Matthew Robin, Sarah Glynn, Ruba Benini, Monica Aleman

**Affiliations:** ^1^ Equine Veterinary Medical Center Member of Qatar Foundation Doha Qatar; ^2^ Division of Pediatric Neurology Sidra Medicine Doha Qatar; ^3^ College of Veterinary Medicine Purdue University West Lafayette Indiana USA; ^4^ Department of Medicine and Epidemiology School of Veterinary Medicine, University of California Davis California USA; ^5^ Present address: Precision Therapy Mazy Belgium

**Keywords:** electroencephalogram, epilepsy, paroxysmal, photic, sedation, seizures

## Abstract

**Background:**

A standardized protocol for electroencephalography (EEG) under standing sedation for the investigation of epilepsy in foals is needed.

**Hypothesis/Objectives:**

To evaluate a modified standardized EEG protocol under standing sedation using sublingual detomidine hydrochloride in Egyptian Arabian foals.

**Animals:**

Nineteen foals (controls, 9; juvenile idiopathic epilepsy [JIE], 10).

**Methods:**

Descriptive clinical study. Foals were classified as controls or epileptic based on history or witnessed seizures and neurological examination. Foals were sedated using sublingual detomidine hydrochloride at a dosage of 0.08 mg/kg to avoid stress associated with injectable sedation. Once foals appeared sedated with their heads low to the ground and with wide base stance (30 minutes), topical lidocaine hydrochloride was applied at the determined locations of EEG electrodes. Fifteen minutes were allowed for absorption and electrodes were placed, protected, and EEG recording performed.

**Results:**

Level of sedation was considered excellent with no need of redosing. The EEG recording lasted from 27 to 51 minutes and provided interpretable data. Epileptic discharges (ED) were noted predominantly in the central‐parietal region in 9 of 10 epileptic foals. Photic stimulation triggered ED in 7 of 10 epileptic foals and in none of the controls. Foals were not oversedated and recovered uneventfully.

**Conclusions and Clinical Importance:**

Sublingual detomidine hydrochloride is a safe, painless, simple, and effective method of sedation for EEG recording in foals. Sublingual sedation allowed the investigation of cerebral electrical activity during states of sleep and arousal, and during photic stimulation for the investigation of epilepsy in foals.

**Abbreviations**
ECG: electrocardiogram
ED: epileptic discharges
EEG: electroencephalogram
JIE: juvenile idiopathic epilepsy
PD: photic driving
SWS: slow wave sleep

## 1 INTRODUCTION

Seizures and epilepsy in horses are reported infrequently in veterinary medicine compared to other species possibly reflecting lower occurrence in equids.^1^, ^2^, ^3^, ^4^, ^5^, ^6^, ^7^ Therefore, limited information is available about etiology, treatment, and outcome.^1^ Attempts have been made to define and classify types of seizures and epilepsy based on clinical descriptions in a limited number of horses.^1^, ^8^ The validity of electroencephalography (EEG) as a diagnostic indicator of intracranial disease was reported based on recordings obtained under general anesthesia for most horses (16 of 20 and 56 of 63 horses from 2 studies, respectively).^1^, ^9^ However, general anesthesia precludes investigation of cerebrocortical activity during states of arousal and sleep essential for studying epileptic disorders, prevents using some epileptic activation procedures, might obliterate paroxysmal discharges that support the diagnosis, and might cause burst suppression depending on the anesthetic agent used.^2^, ^10^, ^11^, ^12^ Therefore, performing EEG under general anesthesia might interfere in the investigation and classification of horses with behavior, sleep, and seizure disorders.

Juvenile idiopathic epilepsy (JIE) is a well‐characterized epileptic disorder based on clinical, neurologic, EEG, and histopathological examination in Egyptian Arabian foals.^2^ It is a self‐limiting disorder with an early onset of seizures ranging from 2 days to 6 months of age, with apparent resolution within 1 to 2 years of life with no known long‐term neurologic sequelae.^2^ Prognosis for life and athletic performance generally is good provided no life‐threatening complications resulting from trauma occur.^2^ Typically, JIE manifests as sudden generalized tonic‐clonic seizures with loss of consciousness followed by cortical blindness, disorientation, obtundation, alterations in behavior, dysphagia, and abnormal gait as common postictal signs.^2^ Affected foals are clinically and neurologically normal during the interictal period.^2^ However, performing EEG during this period has provided essential information that led to the current understanding of JIE in foals.^2^

Electroencephalography is an essential diagnostic modality to define, classify, and advance understanding of pathophysiology, and to improve treatment of epileptic disorders in veterinary medicine as well as in human medicine.^13^, ^14^ Similar to some epileptic syndromes in infants, JIE in foals has a familial basis and is suspected to be inherited in an autosomal dominant manner with a self‐limiting pattern.^2^, ^15^, ^16^ Accurate definition of phenotype must include EEG data obtained and interpreted by a trained neurologist specialized in the investigation of epileptic disorders, especially for those disorders with suspected genetic etiology.^17^ Although sedation protocols for EEG have been published for equids including foals^2^, ^10^, ^18^; a more efficient, simple and less stressful standardized sedation for specific use in foals with epileptic disorders is lacking. Therefore, we sought to evaluate a modified standardized EEG protocol under standing sedation using sublingual detomidine hydrochloride in Egyptian Arabian foals.

## 2 MATERIALS AND METHODS

### 2.1 Foals

The study included 19 foals of Egyptian Arabian breed between the ages of 1.5 and 12 months, 9 colts and 10 fillies. Foals were classified as healthy or epileptic based on history of presence (witnessed or recorded using video surveillance) or absence of seizures, and physical and neurological examination findings. Neurological examination was performed before each EEG study by board‐certified large animal internal medicine specialists. The foals under study were housed alone or with their dams in a padded stall and the study was performed at the Equine Veterinary Medical Center (EVMC) in Doha, Qatar in 2022. The protocol was approved by the Institutional Animal Care and Use Committee of the EVMC (#2021‐1179).

### 2.2 Sedation protocol

Foals were sedated using sublingual detomidine hydrochloride (Domosedan gel 3 mL, 7.6 mg/mL, NJ, USA) at a dosage of 0.08 mg/kg. Adequate level of sedation was determined to be when foals adopted a head position with ears below the level of the withers, wide‐based stance, and apparent somnolent state with minimal reactivity to external stimuli. Once foals appeared sedated within 30 minutes, the specific location for the placement of EEG electrodes was determined by using a flexible ruler and marked with a surgical skin pen (Medline, USA). Local anesthetic cream (Xylocream: 2.5% lidocaine and 2.5% of prilocaine, 30 g, Verisfield, Chalandri, Greece) was applied and massaged into the skin using a glove, and absorption was allowed for 15 minutes before electrode placement in the identified locations. Forty‐five minutes elapsed from the time of sedative administration to the placement of SC needle electrodes and beginning of EEG recording.

### 2.3 Electroencephalogram

A Nicolet NicOne digital EEG system (Natus, Medical, Inc, Middleton, WI, USA) equipped with integrated photostimulator and video was used for the recording of cerebrocortical activity. Electrode nomenclature and placement were based on a modified 10‐20 system used in humans and described in horses previously.^2^, ^10^, ^19^ Number designation followed the EEG protocol used in humans with even and odd numbers denoting the right and left sides, respectively, and vertex denoted as “z.” Disposable subdermal needle electrodes (Neurodart, 0.40 × 13 mm, 250 cm cable, DIN4802, Color set B, SPES Medica, Genoa, Italy) were previously labeled with stickers as follows: Z = ground (1 electrode), FP = frontal polar (2: FP1, FP2), F = frontal (3: F3, Fz, F4), C = central (3: C3, Cz, C4), P = parietal (3: P3, Pz, P4) and O = occipital (2: O1, O2) regions, A = auricular (1 on each ear at its base: A1, A2), OD = right eye (1), and OS = left eye (1).

**FIGURE 1:**
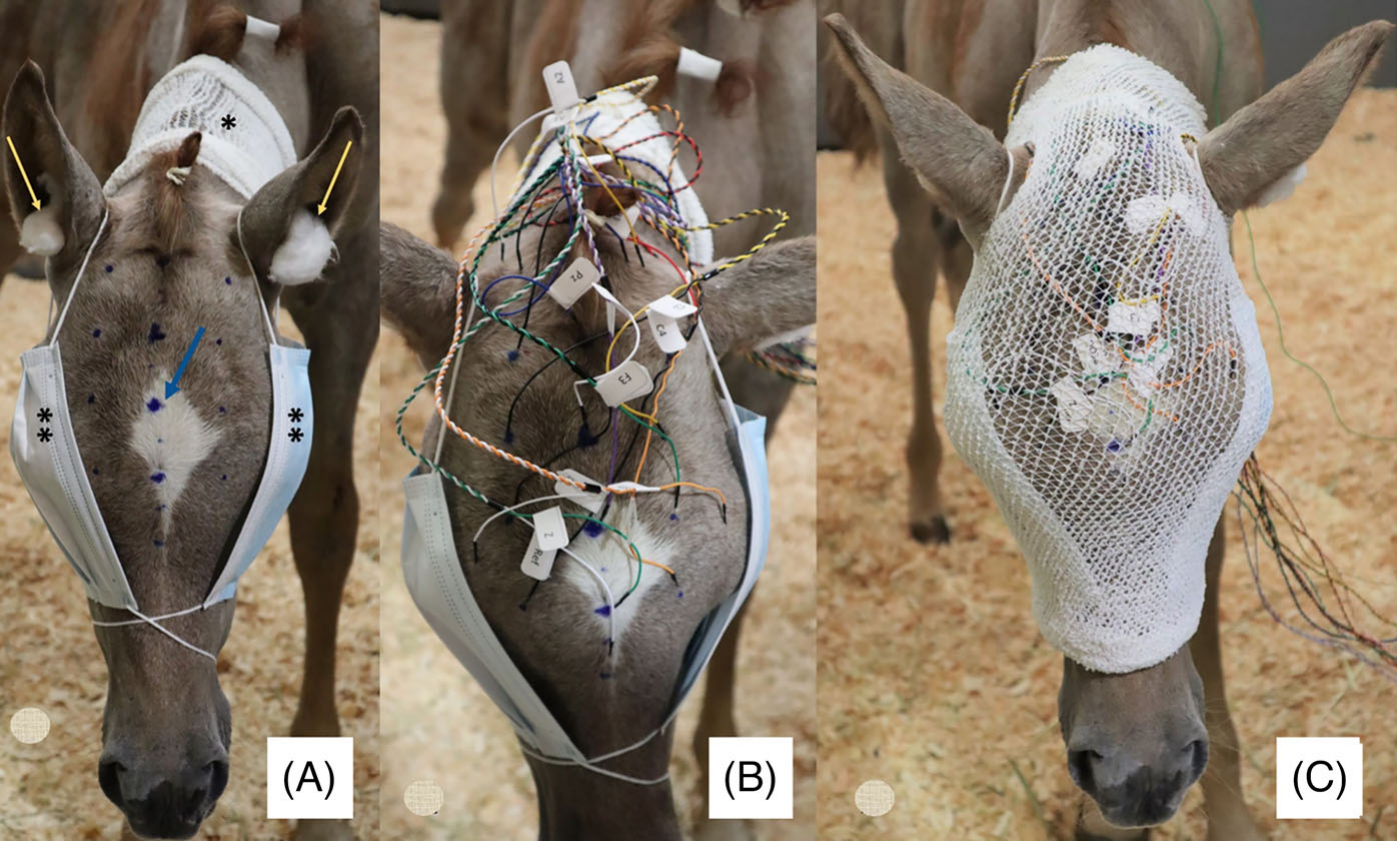
Electrodes placement protocol. (A) Location of electrodes marked with a washable pen (see marks on foal's scalp). *Tubular bandage mesh placed around the neck area to cover and secure the electrodes during recording. Yellow arrows indicate the positioning of the cotton wool balls in the ears to block external noise. Blue arrow indicates the proposed placement of the electrode. **Nonsterile disposable face masks to cover both eyes to avoid visual stimulation during the recording. (B) Placement of disposable subcutaneous needle electrodes. Each electrode was previously labeled with stickers to facilitate its identification for insertion. (C) Tubular bandage mesh covering the electrodes.

The electrodes were placed SC in the scalp located equidistant to each other at approximately 2 cm in cranial to caudal and transverse directions. A soft ruler was used to determine the location of the electrodes, which were marked with a washable pen (Figure 1). The frontal polar electrodes were located at the level of the eye, followed by the frontal electrodes 2 cm caudally, central electrodes 2 cm caudally, and parietal and occipital electrodes in the same manner. A bipolar montage (rostral to caudal and transverse) was used, and for EEG acquisition bandwidth was 0.053‐500 Hz and sampling rate was set at 256 Hz. The electrodes were plugged into an amplifier, and connected to the EEG machine. The impedance was checked at the beginning of each recording to ensure interelectrode impedances were <10 KOhms to minimize recording of artifacts.

Once all of the electrodes were placed, a tubular mesh bandage was placed to cover the foal's head and protect the electrodes and prevent dislodgement of the electrodes by sudden head movements. Holes were cut in the mesh for the ears. Cotton was inserted in the ears and disposable face masks (Xtream Cure & Care, ON, Canada) were used to cover the eyes and minimize environmental sound and visual stimulation while recording the EEG (Figure 1). The amplifier was carried by the handler to stay near the foal and EEG recorded. Movement artifacts including head shaking, snorting, eye movements and ear twitching and were annotated during the recording. The initial EEG recording consisted of a sedated sleep phase. Slow wave sleep (SWS) was determined by the presence of vertex sharp waves, sleep spindles, and K‐complexes.^10^ After recording during sleep, the foal was woken up by gently removing the face mask to allow vision and stimulate the foal. Afterwards, the protocol was followed by 10 minutes of wake recording to evaluate the EEG background during wakefulness.

#### 2.3.1 Activation procedures

**FIGURE 2:**
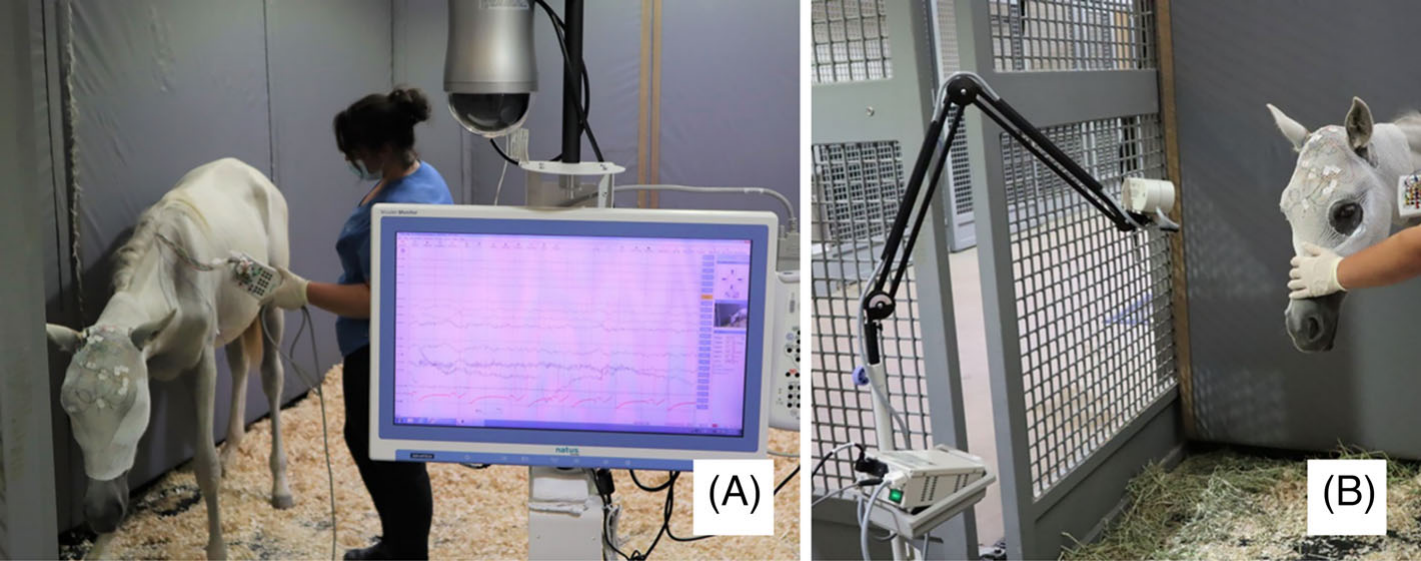
Electroencephalogram instrumentation. (A) EEG setup. (B) Photic stimulation. Note eyes were uncovered for photic stimulation.

Activation procedures were performed during the wake recording including 5 minutes of intermittent photic stimulation and 5 minutes of noise stimulation. Intermittent photic stimulation consisted of 10 second epochs of photic stimulation at different frequencies including 1, 3, 6, 9, 12, 15, 18, 21, 24, 27, 30, 50, and 60 Hz. The foal's head was raised and positioned at a distance of at least 30 cm from the photic stimulator during the intermittent photic stimulation (Figure 2). Lastly, the ears were uncovered for sound stimulation by a loud clap. Uncommonly, foals with JIE might display seizures when stimulated by sound, and therefore this possibility was investigated. Once the recording was completed, electrodes were gently removed and the area was cleaned with alcohol.

#### 2.3.2 EEG interpretation

For review of the EEG tracings, a high frequency filter set between 35 and 70 Hz and low frequency filter at 0.5 Hz were used. Sensitivity ranged from 7 to 10 uV/mm and time scale was 30 mm/s. The bipolar transverse montage was the optimal montage used for review of the EEG tracings, complemented with the average or referential montages to help with localization of epileptic potentials.

## 3 RESULTS

### 3.1 Foals

Based on history, physical and neurological examination, 9 and 10 of 19 foals were classified as controls and epileptic, respectively. The healthy control group included 4 colts ranging in age from 2 to 12 months old, and 5 fillies ranging in age from 2 to 6 months old. The epileptic group consisted of 5 colts (2‐8 months old) and 5 fillies (1.5‐9 months old). Six of 10 foals with epilepsy had generalized onset seizures, 1 had focal onset, and 3 had unknown onset with recorded postictal signs such as blindness, obtundation, tongue paresis, and ataxia a few days to a few weeks before the EEG study. Seizures were characterized as violent generalized tonic‐clonic with facial motor component, trismus, and loss of consciousness. Focal seizures consisted of facial motor alterations including trismus, chewing, tongue paresis, and progression to generalized tonic‐clonic seizures. Postictal signs included disorientation, obtundation, cortical blindness, decreased palpebral reflex, dysphagia, tongue protrusion, proprioceptive deficits of all limbs, and ataxia. Duration of postictal signs varied from a few minutes to days to 1 week (cortical blindness). Otherwise, foals were neurologically normal during the interictal period. Common complications included corneal ulceration, head trauma that in 1 case that resulted in multiple comminuted skull fractures, and dysphagia.

### 3.2 Sedation

Oral sedation was considered excellent for the purpose of the study and did not require redosing. A time of 45 minutes was needed for the level of sedation required (i.e., preventing movement or struggling) for placement of the SC needle electrodes and recording.

### 3.3 Electroencephalogram

Historically, 9 of 10 foals were receiving antiseizure medication as single (phenobarbital, 2; levetiracetam, 2; gabapentin, 1) or multidrug therapy (levetiracetam/phenobarbital, 4). Antiseizure medication was discontinued in the 3 oldest foals (ages 7, 8, and 9 months old) days to weeks before the EEG study. Therefore, at the time of the EEG study, only 6 of 10 foals were receiving antiseizure medication. Timing of the EEG from the last seizure ranged from 6 days to 6 months (median, 1 month). The data obtained was thoroughly examined from EEG tracings and simultaneous video recording for each foal under study. Total duration of EEG recording ranged from 27 to 51 and 40 to 47 minutes in controls and epileptic foals, respectively, and included all phases of vigilance with sleep at the beginning (because of sedation) and wakefulness at the end of the recording.

#### 3.3.1 EEG background during wakefulness and sleep

**FIGURE 3:**
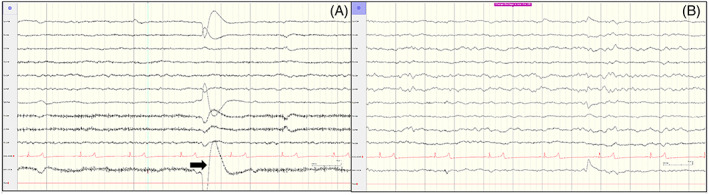
EEG in healthy foal. (A) EEG during wakefulness. Note the larger wave representing eye movement artifact (arrow). (B) EEG during sleep. Note slowing of waves. Calibration bar shown at bottom right of figures.

In all controls as well as in most affected foals (9 of 10), the background during wakefulness was normal and characterized by medium amplitude mixed alpha (8‐9 Hz) and theta (6‐7 Hz) frequencies, seen predominantly in the parasagittal regions (Figures 3A). During sleep, slowing of the waves was noted (Figure 3B) and the background consisted predominantly of intermittent 3‐4 Hz delta activity of slow wave sleep. Vertex sharp waves during sleep were seen at Cz, at times with diffusion to the left and right central regions. Occasional sleep spindles as well as K‐complexes also were recorded over the frontal‐central regions. During the sleep phase, 2nd degree atrioventricular (AV) block was recorded in 11 of 19 foals (controls, 4 of 9; epileptic, 7 of 10). Transient artifacts observed during recording included eye movement, head bobbing, snorting, and chewing, but these artifacts did not preclude the overall acquisition of interpretable data (Figure 3A).

#### 3.3.2 Interictal epileptic abnormalities

**FIGURE 4:**
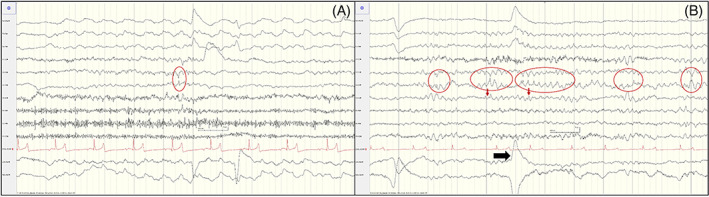
EEG in foals with JIE. (A) EEG of foal A showing focal ED at voltage maximum Cz (circled). (B) EEG of foal B showing multiple ED with voltage maximum at CZ (circled) and spreading (narrow arrows). Note eye movement (thick arrow). Calibration bar shown at bottom center of figures.

Epileptiform discharges (ED) were seen in most affected foals (9 of 10) in the form of either focal, multifocal, or generalized spike and wave discharges (Figure 4). Focal ED were most common (8 of 9), and predominantly in the central vertex (Cz) with diffusion into the fronto‐central or centro‐parietal regions on either hemisphere. Secondary generalization of ED was noted in 3 of 9 JIE foals. Epileptic discharges were seen predominantly during sleep but also during wakefulness in 7 of 9 affected foals. Ictal recordings were not obtained in any of the foals, which was not unexpected considering the short duration of the recording.

#### 3.3.3 Activation procedures

**FIGURE 5:**
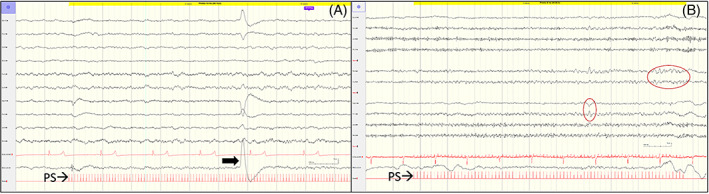
EEG during photic stimulation. (A) Photic driving at 10 Hz in a healthy foal. Large wave represents eye movement (arrow). (B) Epileptic discharges (circled) at 8 Hz shown predominantly at Cz in a foal with JIE. PS = photic stimulation. Calibration bar shown at bottom right of figures.

Auditory stimulation via a loud clap did not result in any changes on the EEG tracing in either control or affected foals. Intermittent photic stimulation resulted in either no response (1 of 10), normal photic driving (2 of 10), abnormal photoparoxysmal responses in the form of increased ED during stimulation (6 of 10), and mixed ED and photic driving (1 of 10). Photoparoxysmal discharges were not associated with clinical manifestations while recording in foals with JIE. Photic driving was seen in 7 of 9 controls. Examples of photic responses are shown on Figure 5.

## 4 DISCUSSION

We found that sublingual detomidine hydrochloride provided an excellent plane of sedation for the duration required for foal preparation and performance of video EEG with additional activation procedures. This protocol proved to be simple, safe, and likely less stressful for the foals than when using IV sedation. Furthermore, sublingual detomidine induced SWS, which facilitated observation of ED in foals with JIE and helped confirmation and classification of foals as epileptic. This sedation protocol proved to be simple, apparently painless, and provided interpretable EEG data in foals, essential for proper phenotyping for future genetic studies.

Our study describes a noninvasive, easy, practical, low cost, and efficient standardized protocol for sedation and EEG for the investigation of epilepsy in foals. Also, this protocol allowed us to minimize the amount of personnel required for foal restraint. It is essential for the recording and interpretation of EEG to avoid movement artifacts associated with struggling. Placement of multiple SC needle electrodes for the recording of EEG can result in struggling. Therefore, an effective sedation protocol was imperative. Detomidine hydrochloride, an α2‐adrenergic agonist, is known for its cardiovascular and respiratory effects such as decreased heart rate, increased occurrence of atrioventricular block, decreased cardiac output, increased or decreased mean arterial pressure in a dose dependent manner, and decreased respiratory rate.^20^, ^21^ Detomidine has been used IV safely in foals and adult horses for EEG recording.^2^, ^18^ However, we wanted to minimize the stress associated with IV injection. Therefore, we elected an alternative route by using sublingual detomidine gel at 0.08 mg/kg in which redosing was not necessary. Based on our previous experience, the manufacturer's recommended dose of 0.04 mg/kg is not adequate for this type of study. Another important factor that helped ensure a proper level of sedation in our study was allowing a minimum of 30 minutes after detomidine administration without any kind of stimulation of the foal. At that time, topical lidocaine hydrochloride was applied and allowed to be absorbed for an additional 15 minutes for a total time of 45 minutes for electrode placement and initiation of the study. Topical lidocaine in the skin facilitated painless needle insertion and did not interfere with the EEG recording as evidenced by visible and interpretable neuronal electrical activity transmitted through the recording electrodes. We considered the level and time of sedation for EEG recording as excellent. Furthermore, the foals did not appear oversedated (e.g., did not collapse or appear unsteady on their feet), and recovered fully with no complications. The procedure was done safely with the foal standing and not requiring anesthesia that might interfere with accurate acquisition and interpretation of EEG data, such as precluding the identification of paroxysmal activity in support of ED.^11^, ^12^, ^18^

Detomidine hydrochloride induced SWS in all 19 foals as described in adult horses.^10^ During this phase, 2nd degree AV block developed in 11 of 19 foals (7 of 10 were epileptic). Development of 2nd degree AV block associated with natural SWS has been documented with concurrent EEG and ECG studies in horses, and is a common normal finding during this state.^10^ Normal findings during SWS in horses, in addition to 2nd degree AV block, include decreased heart and respiratory rates, which also were observed in these foals (data not shown).^18^ These changes might result from an increase in the parasympathetic drive with an associated decrease in cardiac activity and respiratory rate as reported in humans during this stage of sleep.^22^ Sleep can facilitate epileptic activity, and seizures tend to occur during specific states of sleep.^23^ Paroxysmal activity in support of ED was seen during SWS in the majority of affected foals, with the yield of the EEG reaching 90% in our cohort study, which is higher than the 30% to 40% yield reported in the human medical literature.^24^ Only 1 affected foal in our study had a normal EEG. Nevertheless, it is well known that the lack of ED during an EEG recording does not rule out epilepsy.^2^

None of the foals with JIE in our study had obvious preictal signs. Sudden violent generalized tonic‐clonic seizures with loss of consciousness were observed in 6 of 10 foals, and 1 foal had focal facial, oral, and motor seizures that affected the tongue. Three other foals were not observed to have seizures but were found disoriented, obtunded, blind and with tongue protrusion and dysphagia. The postictal phase in foals with observed seizures consisted of disorientation, obtundation, cortical blindness, dysphagia, proprioceptive deficits, and ataxia. The semiology of seizures in these foals with JIE was similar to that previously described for foals with JIE from the United States.^2^

Activation procedures included photic and sound stimulation. Sound stimulation did not trigger ED. Photic stimulation did not result in ED in control foals, whereas ED were triggered in 7 of 10 epileptic foals. Photic stimulation is widely used in routine EEG to aid in the diagnosis of epilepsy in humans.^25^ Paroxysmal discharges were not observed upon photic stimulation in foals with JIE in a previous report.^2^ However, photic stimulation was only performed in a few cases.^2^ Photic driving is a normal response and was observed in a few control (7 of 9) and epileptic foals (3 of 10) in our study. Photic driving is a physiological rhythmic activity representing repetitive visual evoked potentials produced over the occipital region time‐locked in response to a photic flash.^26^ Sound stimulation did not trigger paroxysmal discharges.

Limitations of our study were the low numbers of foals for each group, particularly the control group. Absence of interictal EEG abnormalities does not rule out epilepsy and cases could have been misclassified. Longer duration or repeated EEG recordings in those cases would have been helpful.

## 5 CONCLUSION

We described an easy, low cost, safe, less stressful, and efficient mode to sedate foals sublingually with detomidine hydrochloride for EEG investigation. Sublingual detomidine provided an excellent plane of sedation and sufficient duration of time for EEG recording of interpretable data and performance of activation procedures. Our protocol provided a novel practical standardized process for EEG recording and interpretation which is an essential component for accurate phenotyping in the investigation of epilepsy for future genetic studies. Furthermore, EEG is paramount to classify foals as nonepileptic versus epileptic. Photic stimulation must be included as a routine activation procedure during EEG recording for the investigation of epilepsy because it may trigger ED not detected on routine EEG.

## CONFLICT OF INTEREST DECLARATION

Authors declare no conflict of interest.

## OFF‐LABEL ANTIMICROBIAL DECLARATION

Authors declare no off‐label use of antimicrobials.

## INSTITUTIONAL ANIMAL CARE AND USE COMMITTEE (IACUC) OR OTHER APPROVAL DECLARATION

The protocol was approved by the IACUC of the Equine Veterinary Medical Center (#2021‐1179), Doha, Qatar.

## HUMAN ETHICS APPROVAL DECLARATION

Authors declare human ethics approval was not needed for this study.
